# Supplementary material for: The V-ATPase complex component RNAseK is required for lysosomal hydrolase delivery and autophagosome degradation
Source: Nat Commun. 2024 Sep 5;15:7743. doi: 10.1038/s41467-024-52049-3 (PMC11374810; doi:10.1038/s41467-024-52049-3)
Supplement: Supplementary file 1 — Supplementary Information [file 41467_2024_52049_MOESM1_ESM.pdf]

The V-ATPase complex component RNAseK is required for lysosomal hydrolase delivery and autophagosome degradation

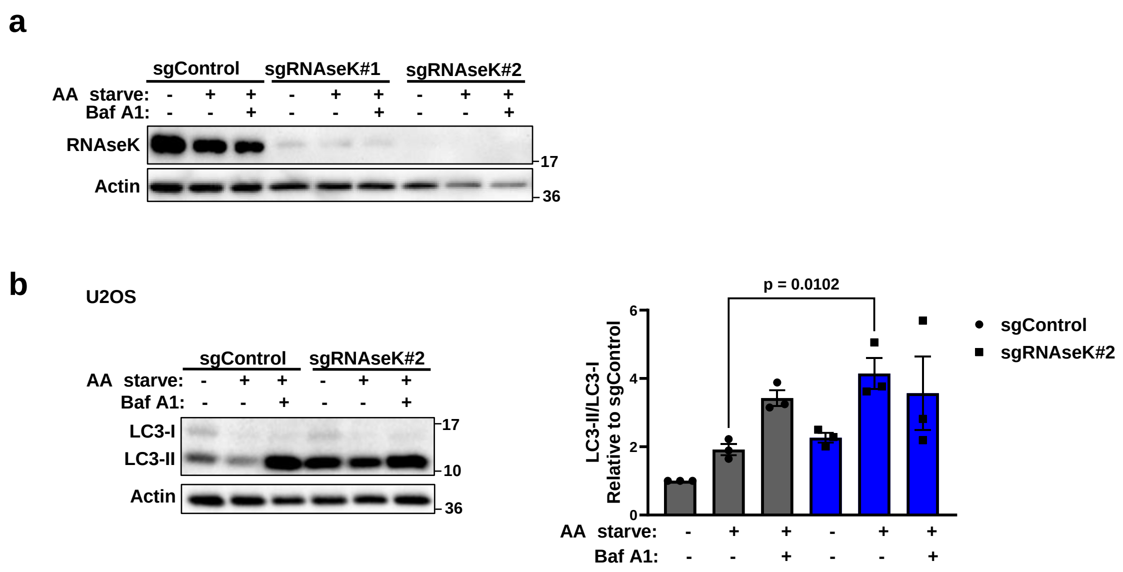

Supplementary Figure 1 (related to Figure 1). Knockout of endogenous RNAseK in cells.

(a) Western blot analyses of endogenous RNAseK in sgControl MEF cells and two RNAseK knockout cells. Cells were either untreated or AA starved in the presence or absence of Baf A1. Molecular weight markers (kDa) are shown. N=1

(b) Western blot analyses of sgControl U2OS cells and RNAseK knockout cells using the indicated antibodies. Cells were left untreated or AA starved for 3 h in the presence or absence of Baf A1. Molecular weight markers (kDa) are shown. N=3. Quantification of LC3-II/LC3-I levels normalised to sgControl is shown on the right. Mean + SEM from three independent experiments, p value was assessed by unpaired Student's *t*-test.

Source data are provided with this paper.

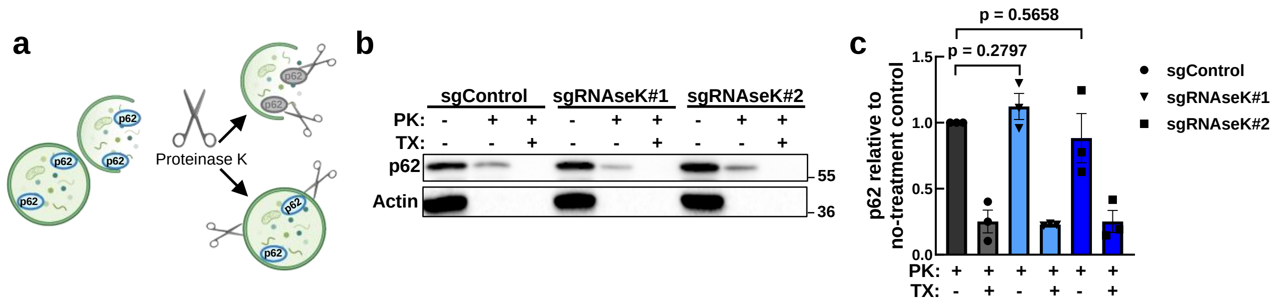

Supplementary Figure 2 (related to Figure 2). Autophagosomes closure is intact in the absence of RNAseK.

(a) Schematic diagram of proteinase K (PK) protection assay. Unclosed autophagosome contents are susceptible to PK degradation while closed autophagosomes contents are protected from degradation. Supplementary figure 2a was created with BioRender.com released under a Creative Commons Attribution-NonCommercial-NoDerivs 4.0 International license https://creativecommons.org/licenses/by-nc-nd/4.0/deed.en.

(b) Western blot analyses of sgControl and sgRNAseK MEF cells cultured in AA starvation media and Baf A1 for 3 h. Cell lysates were incubated with buffer only or with PK in the presence or absence of 0.1% Triton X-100 (TX). Molecular weight markers (kDa) are shown. N=3.

(c) Quantification of (b) with values normalised to buffer only controls. Mean + SEM is shown from three independent experiments, p value is assessed by unpaired Student's t-test.

Source data are provided with this paper.

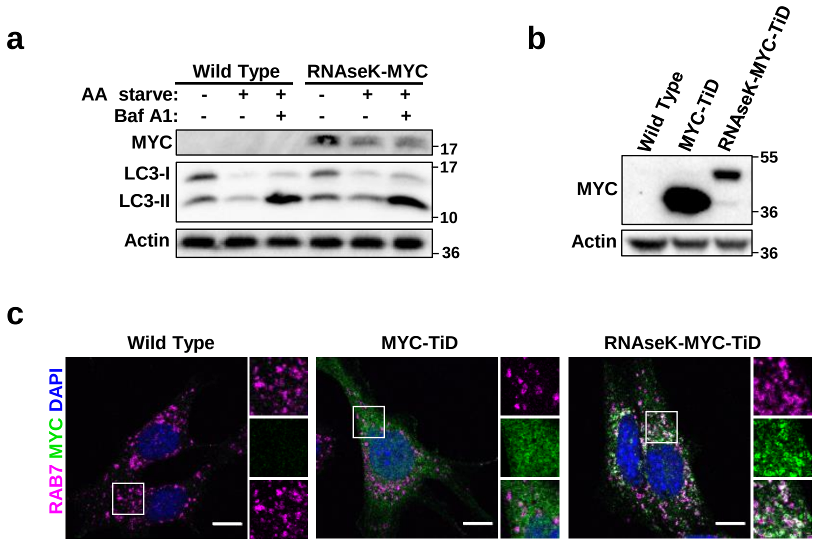

Supplementary Figure 3 (related to Figure 4). Verification of endogenously tagged RNAseK cells and TurboID tagging of exogenous RNAseK.

(a) Western blot analyses of wild type and RNAseK-MYC endogenously tagged MEF cells using the indicated antibodies. Cells were untreated or AA starved for 3 h in the presence or absence of Baf A1. Molecular weight markers (kDa) are shown. N=1.

(b) Western blot analyses of untransfected (wild type), MYC-TurboID (TiD), and RNAseK-MYC-TiD expressing cells using the indicated antibodies. Molecular weight markers (kDa) are shown. N=1.

(c) Representative immunofluorescence images of wild type cells or cells expressing MYC-TiD or RNAseK-MYC-TiD stained using the indicated antibodies. Cells were cultured in the absence of AA for 2 h. Scale bar: 10 µm. N = 1.

Source data are provided with this paper.

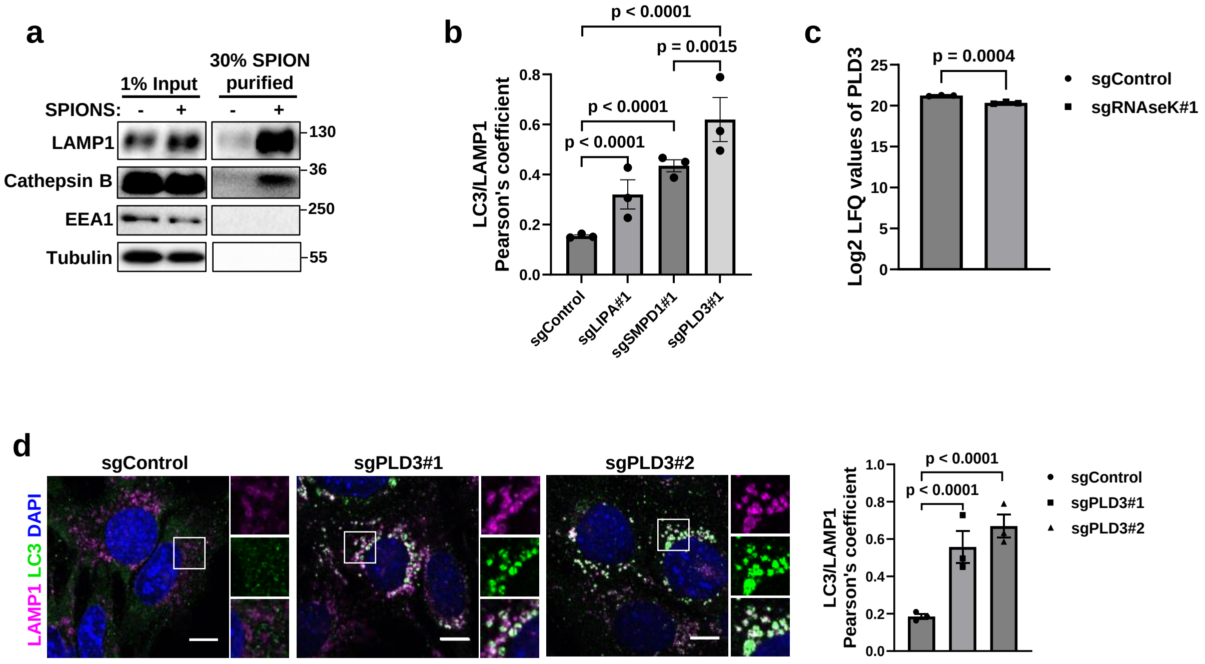

Supplementary Figure 4 (related to Figure 5). Analyses of lysosome-enriched fractions and the role of PLD3 during autophagy.

(a) Western blot analyses of whole cell lysate input and SPION-enriched lysosomal fractions using the indicated antibodies. Molecular weight markers (kDa) are shown. N=1.

(b) CRISPR/Cas9-mediated gene knockout of the indicated lipases in MEF cells. Cells were AA starved, fixed, and stained against endogenous LC3 and LAMP1. Knockout efficiency was assessed by MS analyses. The PCC values of the colocalisation between LC3 and LAMP1 are shown. N=30 cells from 3 independent experiments.

(c) Quantification of PLD3 levels in whole cells lysates derived from MEF cells. N = 1

(d) Representative images of endogenously stained LC3 and LAMP1. PCC quantification is shown on the right. Scale bar: 10 µm. N=30 cells from 3 independent experiments.

In all panels, mean + SEM is assessed by unpaired Student's *t*-test.

Source data are provided with this paper.

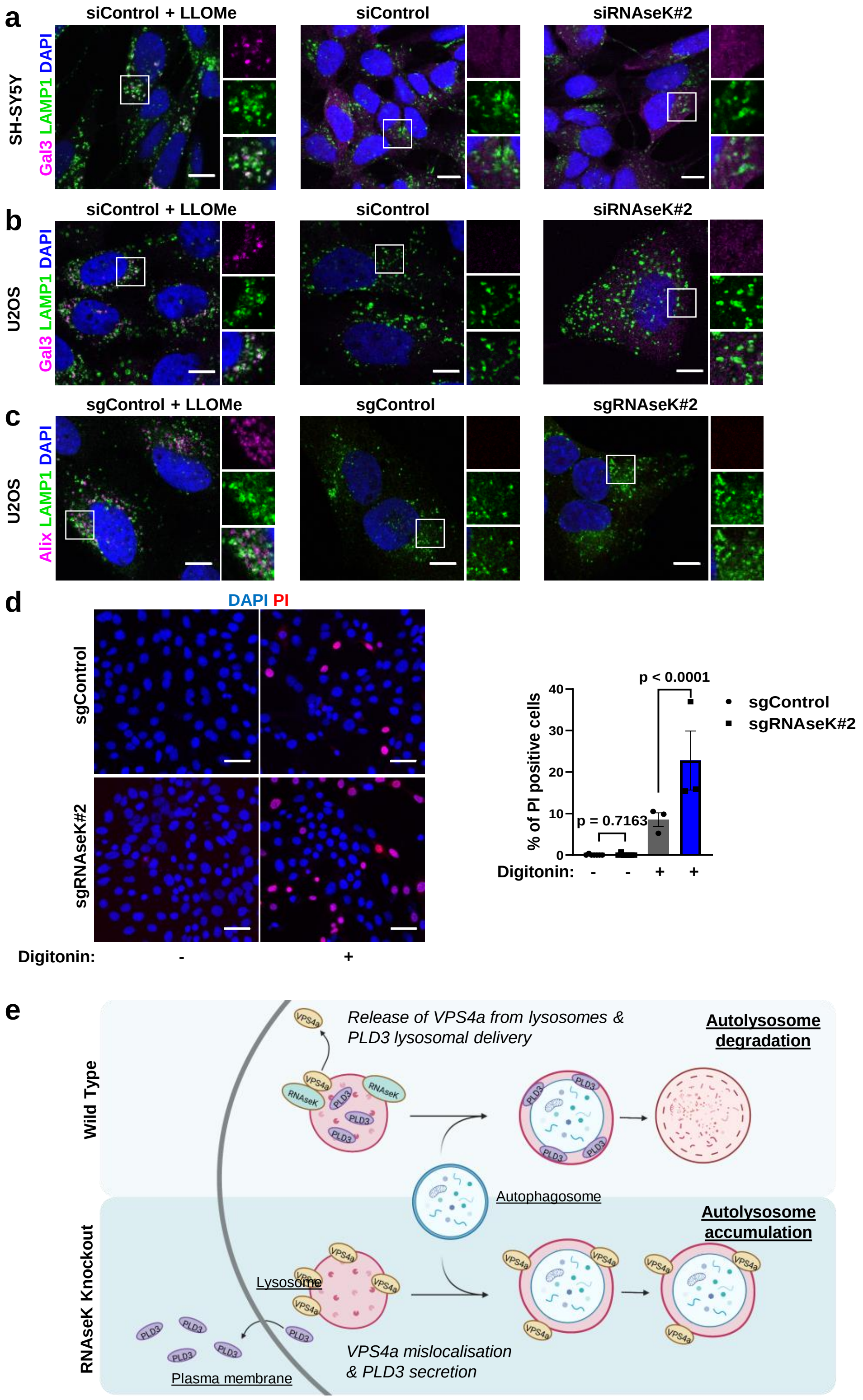

Supplementary Figure 5 (related to Figure 6). No lysosomal damage is detected in the absence of RNAseK.

(a) Representative immunofluorescence images of SH-SY5Y cells transfected with siControl or siPLD3. Where indicated, cells were treated with 0.5 mM LLOMe for 30 min and allowed to recover for an additional 30 min before fixation and staining for endogenous Galectin-3 (Gal3) and LAMP1 antibodies. Scale bar 10 µm. N = 3 independent experiments.

(b) sgControl or sgRNAseK U2OS cells treated as in (A). Scale bar 10 µm. N = 3 independent experiments.

(c) Cells as in (b) stained against endogenous Alix and LAMP1. Scale bar 10 µm. N = 3 independent experiments.

(d) Representative images of digitonin-treated cells used to elicit a plasma membrane damage response. Propidium iodide (PI) and DAPI staining are shown. Quantification of PI-positive cells in sgControl or sgRNAseK is shown on the right. Scale bar 50 µm. Mean + SEM is shown from three independent experiments is assessed by unpaired Student's *t*-test.

(e) Summary depicting a role of RNAseK in autophagosome degradation. In the absence of RNAseK, VPS4a accumulates on lysosomes and leads to enhanced secretion of PLD3 thereby disrupting autophagosome degradation. Supplementary figure 5e was created with BioRender.com released under a Creative Commons Attribution-NonCommercial-NoDerivs 4.0 International license https://creativecommons.org/licenses/by-nc-nd/4.0/deed.en

Source data are provided with this paper.
