# Supplementary material for: The V-ATPase complex component RNAseK is required for lysosomal hydrolase delivery and autophagosome degradation
Source: Nat Commun. 2024 Sep 5;15:7743. doi: 10.1038/s41467-024-52049-3 (PMC11374810; doi:10.1038/s41467-024-52049-3)
Supplement: Supplementary file 3 — Description of Additional Supplementary Files [file 41467_2024_52049_MOESM3_ESM.pdf]

Description of Additional Supplementary Files

File name: Supplementary Data 1
Description: Top hits identified in whole genome CRISPR/Cas9 screen, VPS4a pulldown, and RNAseK TurboID.

File name: Supplementary Data 2
Description: Detailed statistical analyses of hits identified in the whole genome CRISPR/Cas9 screen.

File name: Supplementary Data 3
Description: Detailed protein content in lysosome-enriched fractions derived from wild type or RNAseK knockout MEFs. Log2 fold change values between wild type and sgRNAseK cells and p-values are shown.
