# Supplementary material for: The V-ATPase complex component RNAseK is required for lysosomal hydrolase delivery and autophagosome degradation
Source: Nat Commun. 2024 Sep 5;15:7743. doi: 10.1038/s41467-024-52049-3 (PMC11374810; doi:10.1038/s41467-024-52049-3)
Supplement: Supplementary file 7 — Reporting Summary [file 41467_2024_52049_MOESM7_ESM.pdf]

nature portfolio

Corresponding author(s): Noor Gammoh

Last updated by author(s): May 21, 2024

Reporting Summary

Nature Portfolio wishes to improve the reproducibility of the work that we publish. This form provides structure for consistency and transparency in reporting. For further information on Nature Portfolio policies, see our Editorial Policies and the Editorial Policy Checklist.

Statistics

For all statistical analyses, confirm that the following items are present in the figure legend, table legend, main text, or Methods section.

n/a	Confirmed	
☐	☒	The exact sample size (*n*) for each experimental group/condition, given as a discrete number and unit of measurement
☐	☒	A statement on whether measurements were taken from distinct samples or whether the same sample was measured repeatedly
☐	☒	The statistical test(s) used AND whether they are one- or two-sided<br>*Only common tests should be described solely by name; describe more complex techniques in the Methods section.*
☒	☐	A description of all covariates tested
☒	☐	A description of any assumptions or corrections, such as tests of normality and adjustment for multiple comparisons
☐	☒	A full description of the statistical parameters including central tendency (e.g. means) or other basic estimates (e.g. regression coefficient) AND variation (e.g. standard deviation) or associated estimates of uncertainty (e.g. confidence intervals)
☐	☒	For null hypothesis testing, the test statistic (e.g. *F*, *t*, *r*) with confidence intervals, effect sizes, degrees of freedom and *P* value noted<br>*Give P values as exact values whenever suitable.*
☒	☐	For Bayesian analysis, information on the choice of priors and Markov chain Monte Carlo settings
☒	☐	For hierarchical and complex designs, identification of the appropriate level for tests and full reporting of outcomes
☐	☒	Estimates of effect sizes (e.g. Cohen's *d*, Pearson's *r*), indicating how they were calculated

Our web collection on statistics for biologists contains articles on many of the points above.

Software and code

Policy information about availability of computer code

Data collection	ChemiDoc XRS+ Imaging System (Biorad): western blot chemiluminescence<br>Nikon A1R point scanning confocal: fluorescence imaging<br>Philips/FEI BioTwin CM120 Transmission Electron Microscope: EM analyses<br>StepOne Plus Real-Time PCR System (Applied Biosystems): qPCR<br>BD Area II and BD Fortessa flow cytometer: FACS sorting and analyses<br>Thermo Fusion Lumos: MS analyses of pulldown, TurboID and secreted proteins<br>Orbitrap Fusion Lumos mass spectrometer (Thermo Fisher): for acquisition of lysosomal protein content MS data
Data analysis	ImageJ with EzColocalization plugin: co-localisation analyses<br>ImageLab (Biorad): western blot band quantification<br>FlowJo: flow cytometry analyses<br>Microsoft excel: Volcano plot and qPCR data analyses<br>Perseus: MS data analyses<br>Prism: for statistical analyses and graph presentations<br>Mageck (0.5.6): to count sgRNA and perform statistical analyses for CRISPR/Cas9 screen

For manuscripts utilizing custom algorithms or software that are central to the research but not yet described in published literature, software must be made available to editors and reviewers. We strongly encourage code deposition in a community repository (e.g. GitHub). See the Nature Portfolio guidelines for submitting code & software for further information.

Data

Policy information about availability of data

All manuscripts must include a data availability statement. This statement should provide the following information, where applicable:

- Accession codes, unique identifiers, or web links for publicly available datasets
- A description of any restrictions on data availability
- For clinical datasets or third party data, please ensure that the statement adheres to our policy

Data related to MS analyses of lysosomal protein content has been deposited to ProteomeXchange Consortium via the PRIDE partner repository and can be accessed using the following identifiers:
Project accession: PXD042079
Username: reviewer_pxd042079@ebi.ac.uk
Password: 10EwTAew

Data related to RNAseK-TurboID, VPS4a-pulldown experiments and CRISPR/Cas9 screen are included as supplementary files.

Research involving human participants, their data, or biological material

Policy information about studies with human participants or human data. See also policy information about sex, gender (identity/presentation), and sexual orientation and race, ethnicity and racism.

Reporting on sex and gender	not applicable
Reporting on race, ethnicity, or other socially relevant groupings	not applicable
Population characteristics	not applicable
Recruitment	not applicable
Ethics oversight	not applicable

Note that full information on the approval of the study protocol must also be provided in the manuscript.

Field-specific reporting

Please select the one below that is the best fit for your research. If you are not sure, read the appropriate sections before making your selection.

☒ Life sciences ☐ Behavioural & social sciences ☐ Ecological, evolutionary & environmental sciences

For a reference copy of the document with all sections, see nature.com/documents/nr-reporting-summary-flat.pdf

Life sciences study design

All studies must disclose on these points even when the disclosure is negative.

Sample size	All experiments were conducted 3 times independently unless otherwise stated. A p value of <0.05 was used as a threshold for statistical significance. Sample sizes were determined based on our previous research experience and published findings.
Data exclusions	Data were not excluded except in cases judged to be improperly conducted based on controls (e.g. insufficient gene knockdown or knockout).
Replication	As appropriate, most experiments were repeated in newly generated cells (whether knockout, knockdown or over-expression). Some experiments were reproduced by different lab members at different times.
Randomization	For imaging experiments, images were captured at different parts of the plate.
Blinding	None performed

Reporting for specific materials, systems and methods

We require information from authors about some types of materials, experimental systems and methods used in many studies. Here, indicate whether each material, system or method listed is relevant to your study. If you are not sure if a list item applies to your research, read the appropriate section before selecting a response.

Materials & experimental systems

n/a	Involved in the study	
☐	☒	Antibodies
☐	☒	Eukaryotic cell lines
☒	☐	Palaeontology and archaeology
☐	☒	Animals and other organisms
☒	☐	Clinical data
☒	☐	Dual use research of concern
☒	☐	Plants

Methods

n/a	Involved in the study	
☒	☐	ChIP-seq
☐	☒	Flow cytometry
☒	☐	MRI-based neuroimaging

Antibodies

Antibodies used

β-Actin (Clone AC-74, Sigma Aldrich, A2228); ATG7 (Sigma Aldrich, A2856); Cathepsin B (R&D systems, AF965); CD63 (Clone sc-5275, Santa Cruz, MX-49.129.5); EGFR (Clone sc-03-G, Santa Cruz, 1005); GAPDH (Clone D16H11, CST, 5174); GFP (Chromotek, PABG1-100); GFP (Roche, 11814460001); GM130 (BD biosciences, 610822); HA-Tag (Clone 3F10, Roche, 11867423001); HA-tag (Clone C29F4, CST, 3724); LAMP1 (Abcam, ab25245, for mouse cells); LAMP1 (Iclone D4O1S, CST, 15665, for human cells); LC3B (Sigma Aldrich, L7543); LC3B (Iclone 5F10, Nanotools, 0231-100); MYC-Tag (Clone 9B11, CST, 2276); MYC-Tag (Clone 71D10, CST, 2278s); p62 (Enzo Life Sciences, BML-PW9860-0100); p62 (CST, 5114); PLD3 (Atlas Antibodies, HPA012800); RAB7 (CST, 9364); RFP-tag (Rockland, 600-401-379); EGFR (CST, 2234); Galectin-3 (R&D Systems, AF1197); Ubiquitin (Upstate Cell Signaling Solutions, 07-375); Alix (clone 3A9, CST, 2171S); EEA1 (Clone C45B10, CST, 3288S); Tubulin (CST, 9364); anti-mouse-HRP secondary (CST, 7076); anti-rabbit-HRP secondary (CST, 7074); anti-goat-HRP secondary (Invitrogen, 61-1620); anti-rabbit-Alexa 488 (Invitrogen, A11008); anti-rabbit-Alexa 594 (Invitrogen, A11012); anti-mouse-Alexa 488 (Invitrogen, A1101); anti-mouse-Alexa 594 (Invitrogen, A11032); and anti-rat-Alexa 594 (Invitrogen, A11007); anti-goat-Alexa 594 (Invitrogen, A21469); anti-mouse-Alexa 488 (Invitrogen, A21200)

Validation

- The following antibodies have been validated in this manuscript using genetic knockdown or knockout systems:
ATG7 (Sigma Aldrich, A2856, Fig.1); RNAseK (custom generated, supplementary figure 1); PLD3 (Atlas Antibodies, HPA012800, Figure 4).

- The following anti-tag antibodies were validated in this manuscript using cells that do not express the indicated tag:
MYC-Tag (Clone 9B11, CST 2276) and (Clone 71D10, CST 2278s) (supplementary figure 3);
GFP (Chromotek, PABG1-100) and (Roche, 11814460001) (figure 5);
HA-Tag (Clone 3F10, Roche, 11867423001) and (Clone C29F4, CST, 3724) (figure 2);

- The following antibodies are widely and reliably used for protein detection and are validated by their suppliers:
β-Actin (Clone AC-74, Sigma Aldrich, A2228); GAPDH (Clone D16H11, CST 5174); LC3B (Sigma Aldrich, L7543);

- The following antibodies were validated based on their predicted response to treatment and by their suppliers:
Cathepsin B (R&D systems, AF965) (figure 1);
EGFR (CST, 2234) and EGFR (Clone sc-03-G, Santa Cruz, 1005) (figure 1&4);
p62 (CST, 5114) and p62 (Enzo Life Sciences, BML-PW9860-0100) (figure 2);

- The following antibodies were validated by their manufacturers and exhibited a predicted pattern of staining by immunofluorescence:
CD63 (Clone sc-5275, Santa Cruz, MX-49.129.5); GM130 (BD biosciences, 610822); LAMP1 (Abcam, ab25245, for mouse cells);
LAMP1 (clone D4O1S, CST, 15665, for human cells); RAB7 (CST, 9364); Galectin-3 (R&D Systems, AF1197);

- The following antibody was validated by the manufacturer using none-transfected controls:
RFP-tag (Rockland, 600-401-379);

- The following HRP-conjugated secondary antibodies are widely used for western blot chemiluminescence detection and were diluted at 1:3000:
anti-mouse-HRP secondary (CST, 7076); anti-rabbit-HRP secondary (CST, 7074), anti-goat-HRP secondary (Invitrogen, 61-1620);

- The following fluorescent secondary antibodies are widely used for immunofluorescence staining and were diluted at 1:500:
anti-rabbit-Alexa 488 (Invitrogen, A11008); anti-rabbit-Alexa 594 (Invitrogen, A11012); anti-mouse-Alexa 488 (Invitrogen, A1101); anti-mouse-Alexa 594 (Invitrogen, A11032); and anti-rat-Alexa 594 (Invitrogen, A11007); anti-goat-Alexa 594 (IInvitrogen, A21469); anti-mouse-Alexa 488 (Invitrogen, A21200).

Eukaryotic cell lines

Policy information about cell lines and Sex and Gender in Research

Cell line source(s)

293AAV cells are from Cell Biolabs (#AAV-100)
U2OS, Human neuroblastoma SH-SY5Y and HEK293T cells are from ATCC

Authentication

All cell lines were authenticated by their suppliers.

	Knockout or stably-expressing cells were generated and validated in the corresponding author's lab.<br>Endogenous tagging in cells was confirmed by Sanger sequencing.
Mycoplasma contamination	All cells were routinely tested to ensure they are mycoplasma free.
Commonly misidentified lines<br>(See ICLAC register)	none used

Animals and other research organisms

Policy information about studies involving animals; ARRIVE guidelines recommended for reporting animal research, and Sex and Gender in Research

Laboratory animals	10-12 weeks old male C57Bl6/J mice (Charles River)
Wild animals	none used
Reporting on sex	Male animals were in this study for consistency.
Field-collected samples	none used
Ethics oversight	Home Office (UK)

Note that full information on the approval of the study protocol must also be provided in the manuscript.

Flow Cytometry

Plots

Confirm that:

☐ The axis labels state the marker and fluorochrome used (e.g. CD4-FITC).

☐ The axis scales are clearly visible. Include numbers along axes only for bottom left plot of group (a 'group' is an analysis of identical markers).

☐ All plots are contour plots with outliers or pseudocolor plots.

☐ A numerical value for number of cells or percentage (with statistics) is provided.

Methodology

Sample preparation	Flow cytometry was used to analyse fluorescence intensity in cells labelled with fluorogenic markers of lysosomal activity, including lysosensor Green and Cathepsin B fluorogenic substrate. MEF cells were treated with the fluorogenic substrate for 30 min, trypsinised, resuspended in PBS, and fluorescence signal intensity measured using a BD Fortessa flow cytometer.<br><br>For sorting of CRISPR/Cas9 screen in GFP-LC3 expressing MEFs, cells were amino acid starved, trypsinised, resuspended in PBS, and GFP-positive cells (autophagy-inhibited) were sorted (these data were not included in the manuscript).
Instrument	BD Fortessa flow cytometer (lysosomal function analyses)<br>BD Aria II (GFP-LC3 cell sorting).
Software	FlowJo
Cell population abundance	For experiments involving lysosomal activity, cells were only analysed and not collected.<br><br>For FACS-sorting of CRISPR/Cas9 screen, GFP-positive cells (autophagy-inhibited) were analysed by genomic sequencing and autophagy inhibition was confirmed by the detection of gRNA sequences that target core autophagy machinery (e.g. ATG7).
Gating strategy	Unlabelled cells were used in gating.

☐ Tick this box to confirm that a figure exemplifying the gating strategy is provided in the Supplementary Information.
